# Oligomer β-amyloid Induces Hyperactivation of Ras to Impede NMDA Receptor-Dependent Long-Term Potentiation in Hippocampal CA1 of Mice

**DOI:** 10.3389/fphar.2020.595360

**Published:** 2020-12-10

**Authors:** Ya Wang, Zhaochun Shi, Yajie Zhang, Jun Yan, Wenfeng Yu, Ling Chen

**Affiliations:** ^1^ Department of Physiology, Nanjing Medical University, Nanjing, China; ^2^ Department of Neurology, First Affiliated Hospital of Nanjing Medical University, Nanjing, China; ^3^ Department of Geriatric Medicine, Affiliated Nanjing Brain Hospital of Nanjing Medical University, Nanjing, China; ^4^ Key Laboratory of Endemic and Ethnic Diseases of Education Ministry, Guizhou Medical University, Guizhou, China

**Keywords:** Alzheimer’s disease, amyloid beta, spatial cognition, NMDA receptor, farnesylthiosalicylic acid, Ras-GTP

## Abstract

The activity of Ras, a small GTPase protein, is increased in brains with Alzheimer’s disease. The objective of this study was to determine the influence of oligomeric Aβ_1-42_ on the activation of Ras, and the involvement of the Ras hyperactivity in Aβ_1-42_-induced deficits in spatial cognition and hippocampal synaptic plasticity. Herein, we show that intracerebroventricular injection of Aβ_1-42_ in mice (Aβ-mice) enhanced hippocampal Ras activation and expression, while 60 min incubation of hippocampal slices in Aβ_1-42_ (Aβ-slices) only elevated Ras activity. Aβ-mice showed deficits in spatial cognition and NMDA receptor (NMDAR)-dependent long-term potentiation (LTP) in hippocampal CA1, but basal synaptic transmission was enhanced. The above effects of Aβ_1-42_ were corrected by the Ras inhibitor farnesylthiosalicylic acid (FTS). ERK2 phosphorylation increased, and Src phosphorylation decreased in Aβ-mice and Aβ_1-42_-slices. Both were corrected by FTS. In CA1 pyramidal cells of Aβ_1-42_-slices, the response of AMPA receptor and phosphorylation of GluR1 were enhanced with dependence on Ras activation rather than ERK signaling. In contrast, NMDA receptor (NMDAR) function and GluN2A/2B phosphorylation were downregulated in Aβ_1-42_-slices, which was recovered by application of FTS or the Src activator ouabain, and mimicked in control slices treated with the Src inhibitor PP2. The administration of PP2 impaired the spatial cognition and LTP induction in control mice and FTS-treated Aβ-mice. The treatment of Aβ-mice with ouabain rescued Aβ-impaired spatial cognition and LTP. Overall, the results indicate that the oligomeric Aβ_1-42_ hyperactivates Ras and thereby causes the downregulation of Src which impedes NMDAR-dependent LTP induction resulting in cognitive deficits.

## Highlights

1. Ras activity is increased by *in vivo* or *in vitro* Aβ_1-42_ application.
2. Ras inhibitor FTS relieves Aβ-impaired cognition and NMDAR-dependent LTP.
3. Ras hyperactivation *via* suppressed Src activity impedes NMDAR function.
4. Src inhibitor can impair cognition and LTP in control mice and FTS/Aβ_1-42_ mice.
5. Src activator rescues Aβ-impaired spatial cognition and NMDAR-dependent LTP.

## 1. Introduction

AD is the most frequent cause of dementia in the elderly, featured by progressive loss of memory and amyloid β (Aβ) peptides accumulation in the brain. The injection of synthetic Aβ_1-42_ into the brain or overexpression of Aβ-generating fragments causes learning and memory deficits in various models, indicating a connection between Aβ accumulation and dementia. However, it remains to be clarified how these actions of oligomeric Aβ_1-42_ impair learning and memory, since the memory deficits at early stages of Aβ accumulation are not always associated with massive neuronal degeneration and loss.

High level of Ras, a small GTPase superfamily protein, is found in brains with AD (McShea et al., 2007). Isoprenylated GTPases have been reported to involve in the pathogenesis of AD (Ostrowski et al., 2007; Scheper et al., 2007). Ras interacts with its guanine nucleotide exchange factor to facilitate the conversion of inactive Ras-GDP to active Ras-GTP (Prior and Hancock 2012). Elevated Ras levels were found in early stages of AD (Gartner et al., 1999; Kirouac et al., 2017). The hyperactivation of Ras leads to the disorder of LTP induction (Stornetta and Zhu 2011). The localization of Ras and phosphorylated ERK were increased in plaques and tangles of AD brains (Ferrer et al., 2001; Pei et al., 2002). The Ras-MAPK signaling pathway has been found prior to the formation of plaques and tangles (Gartner et al., 1999) and to influence LTP formation (Ohno et al., 2001). Patients with neurofibromatosis type I (NF1) caused by loss-of-function mutations in the NF1 oncogene have hyperactive Ras, and most NF1 children have cognitive deficits (North et al., 2002). The pharmacological suppression of Ras signaling reverses the deficits in learning in mouse models of NF1 (Li et al., 2005; Cui et al., 2008). However, whether or how hyperactivation of Ras is involved in Aβ_1-42_-induced cognitive deficits remains to be elucidated.

An earlier study reported that tyrosine phosphorylation of NMDA receptors (NMDARs) was enhanced in mice lacking H-Ras (Manabe et al., 2000). Src is a novel H-Ras binding partner. The activation of Ras has been found to modify the downstream effector Src (Thornton et al., 2003). Hippocampal Src kinase activity was increased by H-Ras deficiency, and subsequently enhanced NMDAR function and facilitated LTP induction (Thornton et al., 2003). The administration of farnesyl transferase inhibitor in mice can enhance NMDAR GluN2A/GluN2B phosphorylation through enhanced Src signaling (Chen et al., 2016). Farnesylthiosalicylic acid (FTS), a synthetic Ras inhibitor (Kloog et al., 1999), can dislodge Ras from its anchorage domains to prevent Ras-membrane interactions (Niv et al., 2002). The administration of FTS in mice through enhancing Src activity increases GluN2A/2B phosphorylation (Wang et al., 2018). The inhibition of Ras activity restored normal spine structural plasticity and presynaptic glutamate release in Aβ-treated neurons (Ye and Carew 2010). Therefore, it is speculated that oligomeric Aβ_1-42_ impedes NMDAR-dependent LTP induction via Ras hyperactivity, resulting in cognitive deficits.

The objective of this study was to determine whether oligomeric Aβ_1-42_ affects the activation of hippocampal Ras, and then to investigate how the hyperactivity of Ras is involved in Aβ_1-42_-induced damages in spatial cognition and hippocampal CA1 LTP induction, and to explore the underlying molecular mechanisms. Our results indicate that the oligomeric Aβ_1-42_ causes hyperactivation of Ras, which in turn leads to the downregulation of Src to impede hippocampal NMDAR-dependent LTP induction, resulting in cognitive deficits.

## 2. Materials and Methods

### 2.1. Experimental Animals

All animal experiments complied with the ARRIVE guidelines, and were performed according to the National Institutes of Health guide for the care and use of Laboratory animals (NIH Publications No. 8023, revised 1978). 16-week-old (39.8 ± 1.2 g) and 4-week-old (17.8 ± 1.1 g) male mice (ICR) were in a regulated environment with a 12 h light/dark cycle (lights on at 9:00 A.M.).

### 2.2. Drug Administration

Aβ_1-42_ and 1, 1, 1, 3, 3, 3-hexa-fluoro-2-propanol (HFIP) were purchased from Sigma (St. Louis, United States). The Aβ_1-42_ was dissolved in HFIP and then flash-freezed in liquid nitrogen and lyophilized (Bouter et al., 2013). Lyophilized Aβ_1–42_ was again dissolved in NaOH (100 mM) (Jin et al., 2016). The 16-week-old mice were given the intracerebroventricular (i.c.v.) injection of oligomeric Aβ_1-42_ at the dose of 0.1 nmol/2 μl/side, a non-neurotoxic concentration (Qian et al., 2016). After Aβ_1-42_ injection, the needle was left for over 5 min.

FTS (Cayman chemical, Ann Arbor, USA) was injected (i.p.) at a dose of 3 mg/kg, because this dose of FTS was effective and could enter the brain within 20 to 30 min (Shohami et al., 2003). In *in vitro* experiments, FTS (4.5 μM) could inhibit Ras activity (Kloog et al., 1999), thus this study used five μΜ FTS to treat hippocampal slices.

NMDAR antagonist AP-V, MEK inhibitor U0126, Src-family kinases (SFKs) inhibitor PP2 and the Src activator ouabain (OU) were purchased from Sigma (St. Louis, USA). The slices were treated with PP2 (20 μМ) (Karni et al., 2003), AP-V (20 μМ), U0126 (10 μМ) and ouabain (5 μМ) (Tian et al., 2006). PP2 is reported to pass through the blood-brain barrier in some special animal models (Schumann et al., 2008). However, the injection (i.p.) of PP2 at the dose (0.03 mg/kg) did not alter the levels of NMDAR GluN2A/2B phosphorylation in hippocampus (Sha et al., 2015). In this study, the mice were treated daily with the injection (i.c.v.) of PP2 (1.2 nmol/3 μl per mouse) for eight consecutive days. For repeated injection (i.c.v.), mice were anesthetized with an injection (i.p.) of ketamine (100 mg/kg)/xylazine (10 mg/kg), and placed into a stereotaxic frame (Stoelting). A small hole (2 mm diameter) was drilled in the skull using a dental drill. A stainless steel guide cannula (26-G, Plastics One, Inc., Roanoke, VA) was implanted in the right lateral ventricle (0.22 mm caudal to bregma, 1 mm lateral to the midline, and 1 mm ventral to the pial surface) and anchored to the skull with stainless steel screws and dental cement. On day three after surgery, PP2 was injected using an infusion cannula (30-G) coupled to a motorized injector (Stoelting, Wood Dale, IL, USA) at a rate of 0.5 μL/min. The infusion cannula was left in place for 5 min after injection. Control mice were treated with the injection (i.c.v.) of vehicle (same volume). Ouabain, was administered intraperitoneally at the dose of 1 μg/kg to mice subjected to traumatic brain injury, could improve the neurological function (Dvela-Levitt et al., 2014). In this study, the mice were injected (i.p.) daily with ouabain (1 μg/kg).

### 2.3. Experimental Groups and Sample Size

**FIGURE 1:**
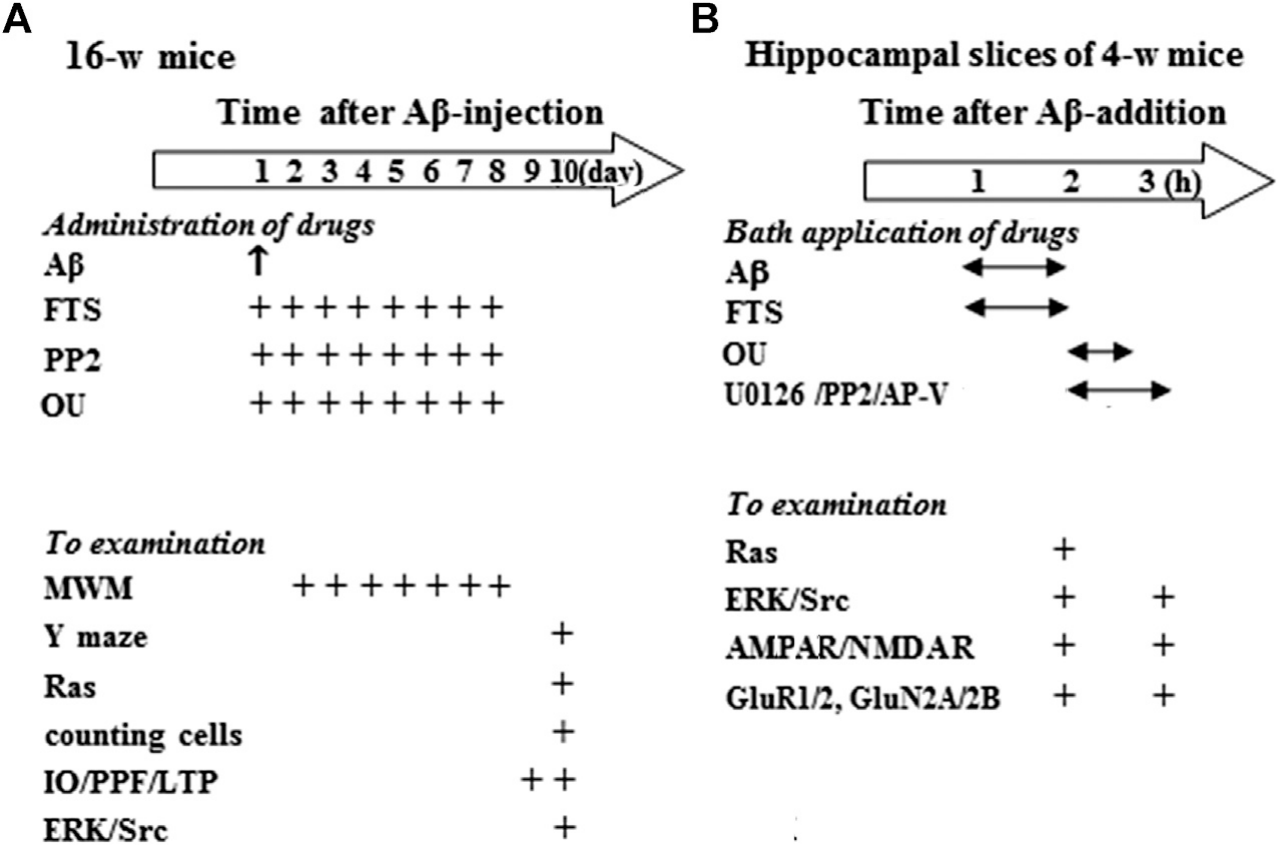
Experimental procedure and group size. **(A)** Hollow arrow shows time (day) after injection (i.c.v.) of Aβ_1-42_. “↑” and “+” indicates the times of drugs administration and examination. MWM: Morris water maze. **(B)** Hollow arrow shows time (h) after Aβ_1-42_ incubation. “↔” and “+” represent the times of Aβ_1-42_ and drugs application, and examination.

Sixteen-week-old mice including control mice (n = 40), FTS-mice (n = 24), Aβ_1-42_-mice (n = 44), Aβ_1-42_/FTS-mice (n = 36), PP2-mice (n = 12), Aβ_1-42_/FTS/PP2-mice (n = 12), ouabain-mice (n = 12) and Aβ_1-42_/ouabain-mice (n = 12) were divided into following groups (Figure 1A). The first group (32 control mice, 20 FTS-mice, 36 Aβ_1-42_-mice, 28 Aβ_1-42_/FTS-mice, eight PP2-mice, eight Aβ_1-42_/FTS/PP2-mice, eight ouabain-mice and eight Aβ_1-42_/ouabain-mice) was used to examine the spatial cognitive behaviors, surviving pyramidal cells, Ras activity and ERK/Src phosphorylation. The second group (8 control mice, 4 FTS-mice, eight Aβ_1-42_-mice, eight Aβ_1-42_/FTS-mice, four PP2-mice, four Aβ_1-42_/FTS/PP2-mice, four ouabain-mice and four Aβ_1-42_/ouabain-mice) was used to examine synaptic transmission and LTP induction.

Hippocampal slices (approximately four to five slices per mouse) obtained from 4-week-old control mice (n = 136) were treated with Aβ_1-42_ and various drugs or vehicle, and then divided into two experimental groups (Figure 1B): The first group was used to examine Ras activities, ERK/Src phosphorylation, expression and phosphorylation of GluR1/2 or GluN2A/2B; the second group was used to examine AMPAR/NMDAR function.

### 2.4. Behavior Analysis

*Morris water maze task* (MWM): A pool (diameter = 120 cm) was prepared with black-colored plastic. The water temperature (22 ± 1 °C) of pool was regulated using a bath heater. For the hidden-platform test, a cylindrical dark-colored platform (diameter = 7 cm) was placed in 0.5 cm below the water surface. The trial was stopped when the mouse did not reach the platform within 90 s. Each mouse started pseudorandomly from one of four quadrants. Four trials with an interval of 30 min were conducted each day. The probe trial was performed after the hidden-platform test at 24 h. A computer with a video camera system was used to record the swimming paths and time. In the hidden-platform test, latency to reach the platform and swimming speed were measured.

*Y-maze task*: The mouse could move freely within 8 min. When the hind paws were completely placed in the arm, arm entry was counted. Alternation was determined as successive entries into the three arms on overlapping triplet sets. The percentage alternation was calculated as the ratio of actual to possible alternations (defined as the total number of arm entries minus two).

### 2.5. Histological Examination and Analysis

After anesthetized with ketamine (100 mg/kg)/xylazine (10 mg/kg, i. p.), the mice were perfused transcardially with 4% paraformaldehyde. The removed brains were postfixed overnight in the 4% paraformaldehyde. The paraffin embedding was processed, and then the coronal sections (5 μm) were cut. The hippocampal sections were stained by toluidine blue. We used a conventional light microscope (Olympus DP70, Japan, × 40) to observe the hippocampal pyramidal cells. For the analysis of cell quantification, every fourth section was stained by toluidine blue. The healthy pyramidal cells was counted throughout hippocampal CA1 regions (n = 10 sections per brain) using the manual tag function of Image Pro-Plus 6 (Media Cybernetics). The density of cells was expressed as cell number per square millimeter (Dawson et al., 2003).

### 2.6. Electrophysiological Analysis

*Slice preparations*: Hippocampal slices were obtained from 16-week-old mice for field potential recording or 4-week-old mice for whole cell patch-clamp recording. The brains were kept in ice-cold and oxygenated ACSF (in mM: NaCl 126, CaCl_2_ 1, KCl 2.5, MgCl_2_ 1, NaHCO_3_ 26, KH_2_PO_4_ 1.25, and D-glucose 20, pH: 7.4) for 10 min. The coronal slices (400 μm) of hippocampus were cut using a vibrating microtome. The slices were recovered for 1 h, and then transferred to a recording chamber of 30 ± 1 °C.

*Field potential recording*: For recording hippocampal CA3-CA1 synaptic properties, a bipolar tungsten electrode was placed in CA1 *str*. radiatum to stimulate Schaffer collateral afferents using a stimulator. Excitatory postsynaptic potential (EPSP) was recorded in CA1 *str*. radiatum by a 4–5 MΩ resistance glass microelectrode. 1) Input/output (I/O) curve: EPSP slopes were plotted against various stimulus intensities (0.1–1.1 mA). 2) Paired-pulse facilitation (PPF) was evoked by two stimuli with inter-pulse interval (IPI) of 50–100 ms. The value of paired-pulse ratio (PPR) = EPSP_S2_/EPSP_S1_ × 100. The EPSP_S1_ and EPSP_S2_ represented the two stimuli-induced EPSP slopes. 3) High-frequency stimulation (HFS, 100 Hz, 1 s) was delivered to induce LTP.

*Cell patch-clamp recording*: AMPA-evoked currents (*I*_AMPA_) and NMDA-evoked currents (*I*_NMDA_) were induced in hippocampal CA1 pyramidal cells using a picospritzer (rapid drug delivery system) and recorded using an EPC-10 amplifier (HEKA Elektronik, Germany) as described (Zhou et al., 2016; Wang et al., 2018). A glass pipette was filled with an internal solution (pH 7.2) (in mM: Cs-gluconate 120, NaCl 2, MgCl_2_ 4, Na_2_-ATP 4, HEPES 10, EGTA 10). The holding potential was kept at -60 mV. *I*_AMPA_ was induced by AMPA (1–300 μM) in the same neuron to produce dose-response curve. To record *I*_NMDA_, the slices were perfused with the oxygenated magnesium-free ACSF. NMDA (1–1,000 μM) was applied to *I*_NMDA_. The AMPAR antagonist CNQX (5 μM) or the NMDAR antagonist AP-V (20 μM) was used to verify the obtained *I*_AMPA_ and *I*_NMDA_.

### 2.7. Western Blot Analysis

The ketamine (100 mg/kg)/xylazine (10 mg/kg, i. p.) were used to anesthetize mice. After the mice were decapitated, the hippocampus were rapidly removed and homogenized in a lysis buffer (in mM: Tris-HCl 50, NaCl 150, EDTA 5, NaF 10, sodium orthovanadate 1, phenylmethylsulfonyl fluoride 1, 1% Triton X-100, 0.5% sodium deoxycholate and protease inhibitor cocktail). After that, total proteins (20 μg) were separated by SDS-PAGE and blotted onto a polyvinilidene fluoride (PVDF) membrane. The membranes were incubated in antibodies of phospho-ERK1/2 (1:1,000; CST, USA); phospho-GluR1, phospho-GluR2, phospho-GluN2A and phospho-GluN2B (1:1,000; Abcam, United Kingdom); phospho-Src (1:1,000; Millipore, USA). After visualization, the membranes were treated with stripping buffer (Restore; Pierce) for 15 min, and then incubated with antibodies of ERK1/2 (1:1,000; CST, USA); GluR1, GluR2, GluN2A and GluN2B (1:1,000; Abcam, United Kingdom); Src (1:1,000; Millipore, USA) or β-actin (1:1,000; CST, USA). Ras activation assay was implemented according to the instructions of the company (Cell biolabs). The small GTPase is tested by Western blot using a target-specific antibody included in the kit.

### 2.8. Data Analysis

SPSS software was used to perform Statistical analyses. The data were expressed as the means ± standard error (SE). Repeated measures ANOVA were used to analyze the behavioral and electrophysiological data. The actual values of the ANOVAs (one-way ANOVA, two-way ANOVA, repeated measures ANOVA) followed by Bonferroni post hoc analysis are shown in the section of Results. Differences at *p* < 0.05 were considered statistically significant.

## 3. Results

### 3.1. Aβ_1-42_ Impairs Spatial Cognition Through Enhanced Ras Activation

**FIGURE 2:**
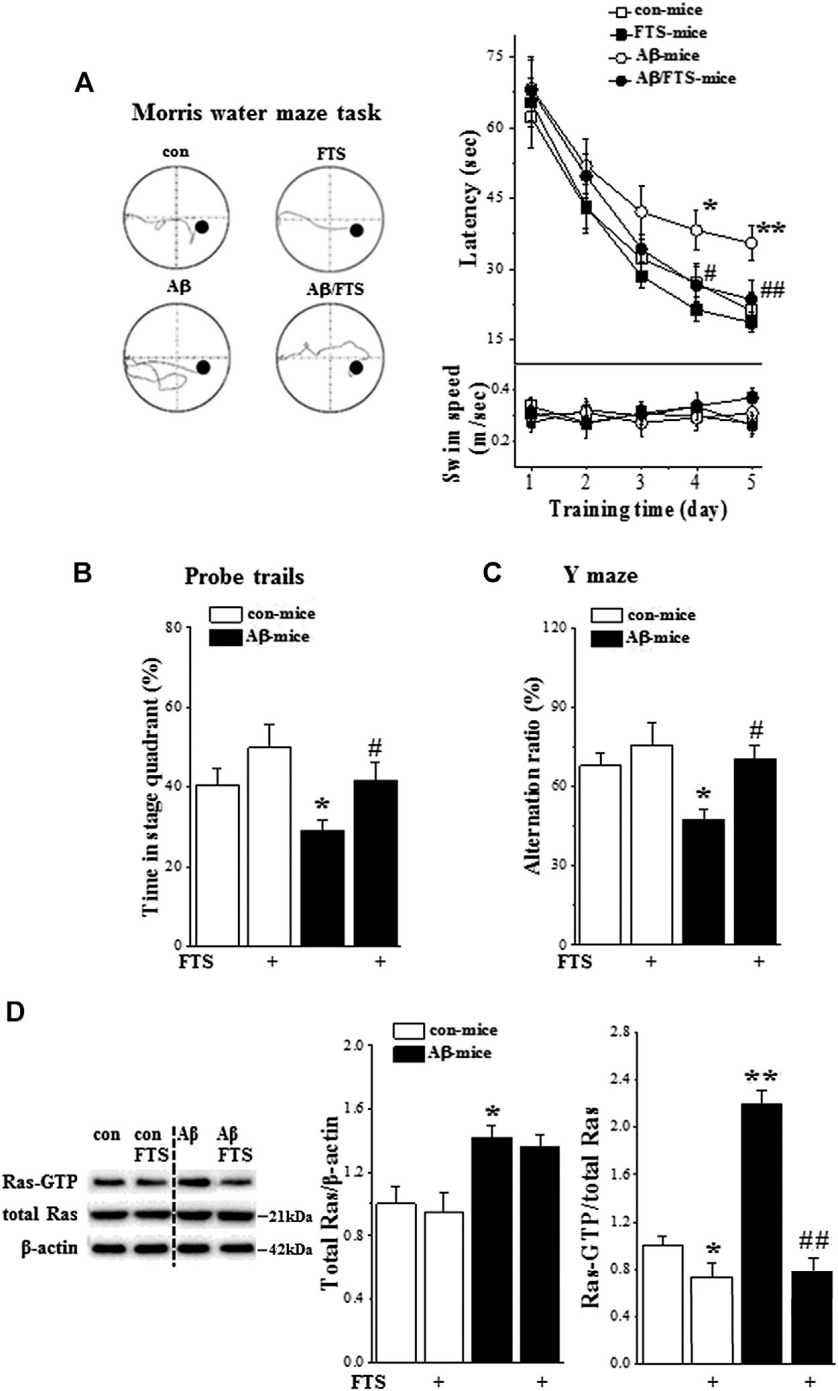
Aβ_1-42_ impairs spatial cognition *via* Ras hyperactivation. **(A)** Each point (upper panel) indicates group mean latency (sec) to reach the hidden-platform in control mice (con-mice), Aβ_1-42_-mice (Aβ-mice), FTS-treated control mice (FTS-mice) and FTS-treated Aβ_1-42_-mice (Aβ/FTS-mice). Each point (bottom panel) indicates mean swim speed (m/sec). Left panels show representative swimming tracks of mice searching for the underwater platform at day 5 after training. *p < 0.05 and **p < 0.01 vs. control mice; #*p* < 0.05 and ##*p* < 0.01 vs. Aβ_1-42_-mice (repeated-measures ANOVA, n = 8 per group). **(B)** Bars present the percentage of time spent in target quadrant of probe trails. *p < 0.05 vs. control mice; #*p* < 0.05 vs. Aβ_1-42_-mice (two-way ANOVA, n = 8 per group). **(C)** Bar graph indicates group mean of alternation rate (%) in Y-maze task. *p < 0.05 vs. control mice; #*p* < 0.05 vs. Aβ_1-42_-mice (two-way ANOVA, n = 8 per group). **(D)** Effects of Aβ_1-42_ on expression and activation of hippocampal Ras. Bar graphs represent the levels of total Ras protein and Ras-GTP in mice. The mean value per experimental group was normalized by control mice. *p < 0.05 and **p < 0.01 vs. control mice; ##*p* < 0.01 vs. Aβ_1-42_-mice (two-way ANOVA, n = 6 per group).

Spatial cognitive behaviors were examined in mice injected (i.c.v.) with Aβ_1-42_ (Aβ-mice) (n = 8 per group; Figure 1A). The repeated measures ANOVA revealed a progressive decline in the escape latency to reach the hidden platform in the MWM with training days in all groups (F_(4, 112)_ = 41.460, *p* < 0.001; Figure 2A). In comparison with control mice, the escape latency was significantly increased in Aβ-mice (F_(1, 14)_ = 13.095, *p* = 0.003) without changes in swimming speed (*p* > 0.05). A probe trial was carried out to measure the swimming time spent in four quadrants. Notably, the swimming time in the target quadrant was reduced in Aβ-mice compared with control mice (*p* = 0.042; Figure 2B). In Y-maze, the alternation ratio in Aβ-mice was lower compared with control mice (*p* = 0.038; Figure 2C).

The Ras-GTP pull-down assay using a Ras effector protein that recognizes the active state of Ras was performed to examine the influence of Aβ_1-42_ on the expression and activity of hippocampal Ras (n = 6 per group). Compared with control mice, the level of Ras protein in Aβ_1-42_-mice was increased by approximately 30% (*p* = 0.049; Figure 2D) and the level of Ras-GTP was elevated at least 2-fold (*p* < 0.001). FTS has been demonstrated to selectively disrupt the interactions of active Ras proteins with the plasma membrane (Elad-Sfadia et al., 2002). The administration of FTS (Figure 1A) not only reduced the level of Ras-GTP in control mice (FTS-mice, *p* = 0.041) but also prevented the increase in Ras-GTP in Aβ_1-42_-mice (Aβ_1-42_/FTS-mice, *p* < 0.001) with no change of Ras protein (*p* > 0.05).

Treatment of Aβ-mice with FTS (Figure 1A) corrected the increase in escape latency in the MWM (*p* = 0.009) and the decline in swimming time in the target quadrant of the probe trial (*p* = 0.032). Subsequently, the alternation ratio in the Y-maze was restored in Aβ_1-42_/FTS-mice (*p* = 0.027). By contrast, FTS-mice did not show changes in the escape latency of the MWM (*p* > 0.05), the swimming time of the target quadrant (*p* > 0.05) and the alternation ratio (*p* > 0.05).

### 3.2. Aβ_1-42_-Activated Ras Enhances Synaptic Transmission and Impairs LTP

**FIGURE 3:**
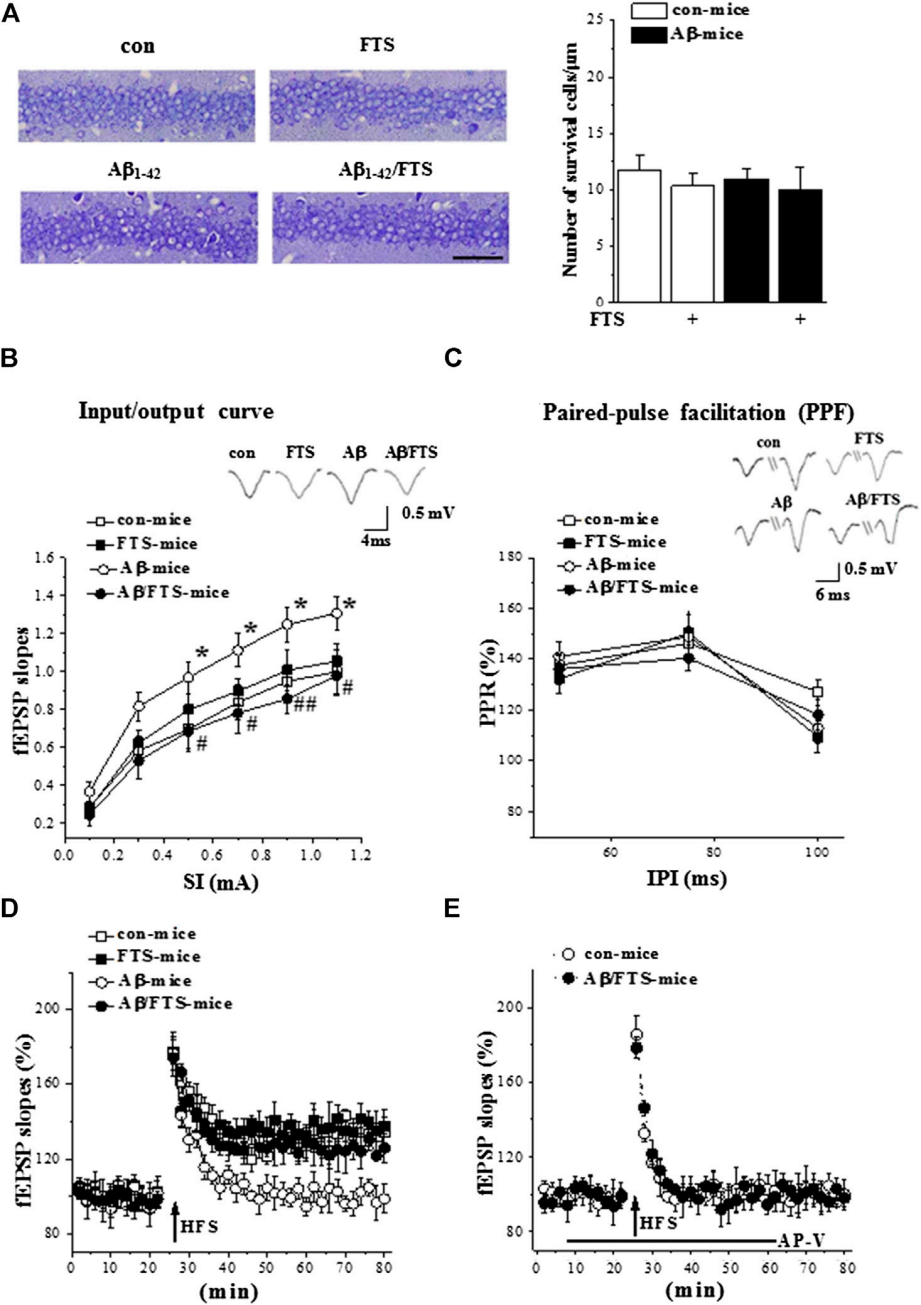
Aβ_1-42_-activated Ras enhances synaptic transmission and impairs LTP. **(A)** Representative hippocampal CA1 images in mice. Scale bars = 50 μm. Bar graph represents the counts of surviving pyramidal cells (two-way ANOVA, n = 6 per group). **(B)** fEPSPs slopes were plotted against intensity of test stimuli from 0.1 to 1.1 mA. Typical traces evoked by a testing stimulation (0.5 mA). *p < 0.05 vs. control mice; #*p* < 0.05 and ##*p* < 0.01 vs. Aβ_1-42_-mice (repeated-measures ANOVA, n = 8 per group). **(C)** Paired-pulse ratios of fEPSP slopes were plotted against interpulse intervals (IPIs) of stimuli from 50 to 100 ms. Typical traces evoked with IPI of 75 ms (repeated-measures ANOVA, n = 8 per group). **(D)** Effects of FTS on Aβ_1-42_-impaired induction of LTP. A solid arrow indicates when HFS was given. **(E)** NMDAR dependency of LTP induction. Black line indicates duration of NMDAR antagonist AP-V applied.

The counts of surviving pyramidal cells (n = 6 per group; Figure 1A) showed that the number of cells was unchanged in the hippocampal CA1 region of Aβ_1-42_-mice (*p* > 0.05; Figure 3A), Aβ_1-42_/FTS-mice (*p* > 0.05), and FTS-mice (*p* > 0.05) in comparison with control mice.

Subsequently, we examined the basal properties of hippocampal CA3-CA1 synaptic transmission and the induction of LTP by field potential recordings (n = 8 slices/4 mice per group). The input-output relationship was built by plotting the slope of filed EPSP(fEPSP) vs. the intensities (0.1–1.1 mA) of stimulating Schaffer collateral-commissural fibers. Compared with control mice, the Aβ_1-42_-mice had increased fEPSP slopes (F_(1,14)_ = 12.624, *p* = 0.003; Figure 3B), which was corrected by FTS treatment (F_(1,14)_ = 18.196, *p* = 0.001). PPF was evoked by 0.5 mA two stimuli (50–100 ms IPI). The PPR of fEPSP slopes was not changed in Aβ_1-42_ mice (*p* > 0.05; Figure 3C) or Aβ_1-42_/FTS mice (*p* > 0.05). The delivery of high-frequency stimuli (HFS, 100 Hz with 100 pulses) in control mice induced an about 40% increase in the fEPSP slopes (at 55–60 min after HFS; Figure 3D), which was blocked by AP-V. The application of the same HFS protocol in Aβ_1-42_ mice did not cause a long-lasting increase in the fEPSP slopes (Figure 3E). NMDAR-dependent LTP could be induced in Aβ_1-42_/FTS mice. The fEPSP slopes (*p* > 0.05) and the PPR value (*p* > 0.05) in FTS-mice did not differ from those in control mice. The amplitude of NMDA-dependent LTP in FTS-mice failed to be increased compared with control mice (Figure 3D).

### 3.3. Aβ_1-42_-Activated Ras Enhances ERK Signaling and Suppresses Src Activity

**FIGURE 4:**
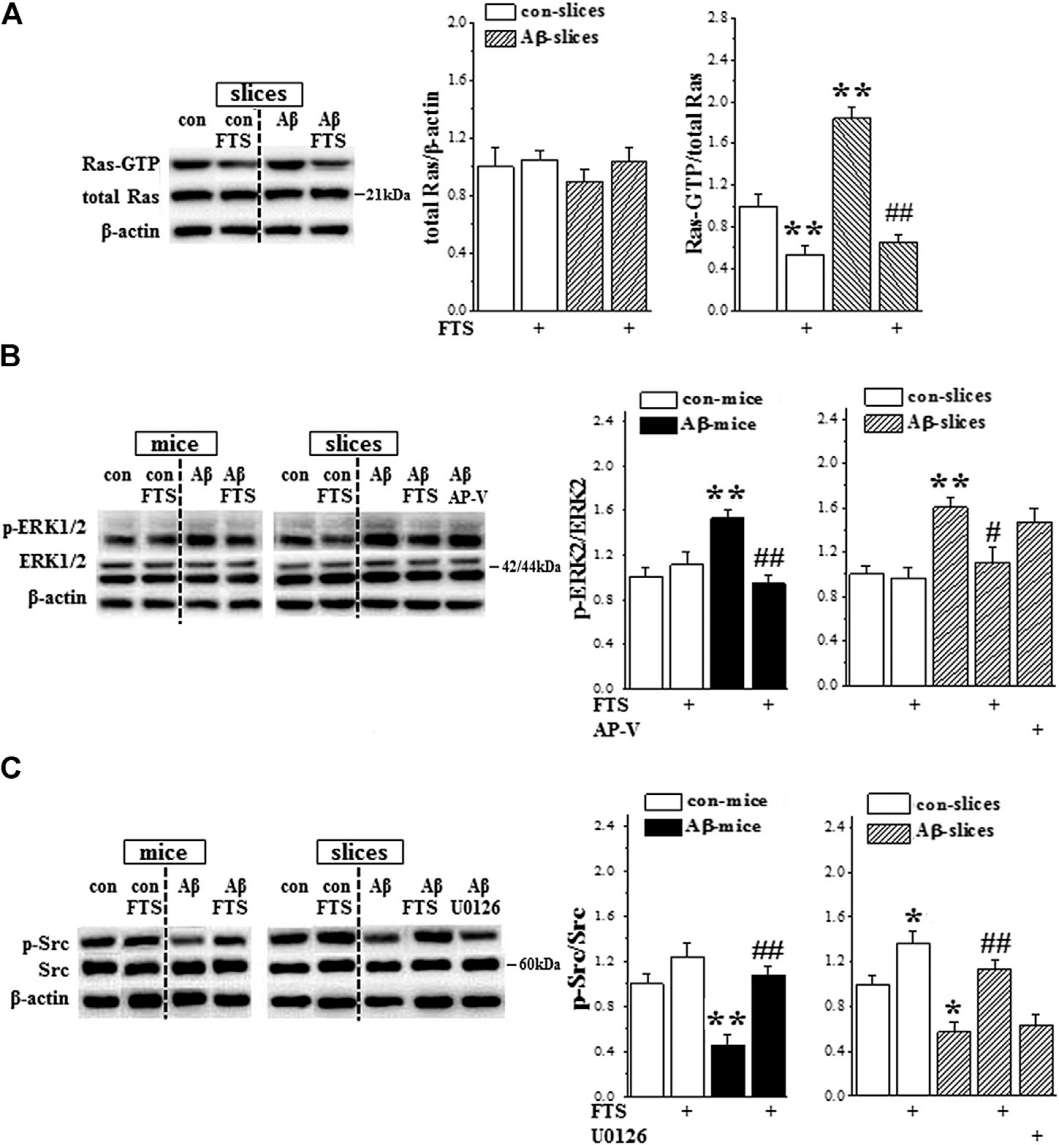
Aβ_1-42_-activated Ras enhances ERK signaling and suppresses Src activity. **(A)** Acute effect of Aβ_1-42_ on hippocampal Ras activity. Left panels show representative pictures of Western blot in control slices (con), FTS-slices (FTS), Aβ_1-42_-slices (Aβ) and Aβ_1-42_/FTS-slices (Aβ/FTS). Bar graphs represent the levels of Ras protein and Ras-GTP. The mean value per experimental group was normalized by control. **p < 0.01 vs. control slices; ##*p* < 0.01 vs. Aβ_1-42_-slices (two-way ANOVA, n = 12 per group). **(B)** and **(C)** Bar graphs show the levels of hippocampal ERK2 and Src phosphorylation (*p*-ERK2, *p*-Src) in control mice/slices (con), FTS-mice/slices (FTS), Aβ_1-42_-mice/slices (Aβ) and Aβ_1-42_/FTS-mice/slices (Aβ/FTS). *p < 0.05 and **p < 0.01 vs. control mice/slices; #*p* < 0.05 and ##*p* < 0.01 vs. Aβ_1-42_-mice/slices (two-way ANOVA, n = 6 mice or 12 slices per group).

Primary neurons treated with oligomeric Aβ_1-42_ for 5 min exhibited a significant increase in Ras-ERK signaling activity (Kirouac et al., 2017). To explore the molecular mechanisms underlying Aβ_1-42_-impaired NMDAR-dependent LTP and Aβ_1-42_-enhanced postsynaptic response by the hyperactivity of Ras, further experiments were conducted to detect the functions of AMPA receptor (AMPAR) and NMDAR. First, the activity of Ras was examined in the hippocampal slices treated with Aβ_1-42_ (Aβ_1-42_-slices), Aβ_1-42_/FTS (Aβ_1-42_/FTS-slices) or FTS (FTS-slices) for 60 min (n = 12 slices/4 mice per group; Figure 1B). In comparison with control, the level of Ras-GTP was significantly increased in Aβ_1-42_-slices (*p* < 0.001; Figure 4A), which was sensitive to the addition of FTS (*p* < 0.001). The level of GTP-Ras was lower in FTS-slices (*p* = 0.008).

Subsequently, we examined the phosphorylation of ERK1/2 (phospho-ERK1/2) and Src (phospho-Src) in the hippocampus of Aβ_1-42_-mice (Figure 1A, n = 6 mice per group) or Aβ_1-42_-slices (Figure 1B, n = 12 slices/4 mice per group). The levels of phospho-ERK2 in Aβ_1-42_-mice (*p* = 0.004; Figure 4B) and Aβ_1-42_-slices (*p* = 0.005) were significantly increased without changes in the levels of ERK2 protein (*p* > 0.05). The increased phospho-ERK2 in Aβ_1-42_-mice (*p* = 0.002) and Aβ_1-42_-slices (*p* = 0.026) was corrected by the application of FTS, but not AP-V (*p* > 0.05). Notably, both Aβ_1-42_-mice (*p* = 0.005; Figure 4C) and Aβ_1-42_-slices (*p* = 0.038) showed a significant decline in the level of phospho-Src, which could be rescued by the application of FTS (mice: *p* = 0.001; slices: *p* = 0.003). In addition, the level of phospho-Src showed a slight increase in FTS-slices (*p* = 0.037), but not in FTS-mice (*p* > 0.05). The levels of phospho-ERK2 were not altered in both of FTS-mice and FTS-slices (*p* > 0.05).

### 3.4. Aβ_1-42_-Activated Ras Signaling Enhances AMPAR Function

**FIGURE 5:**
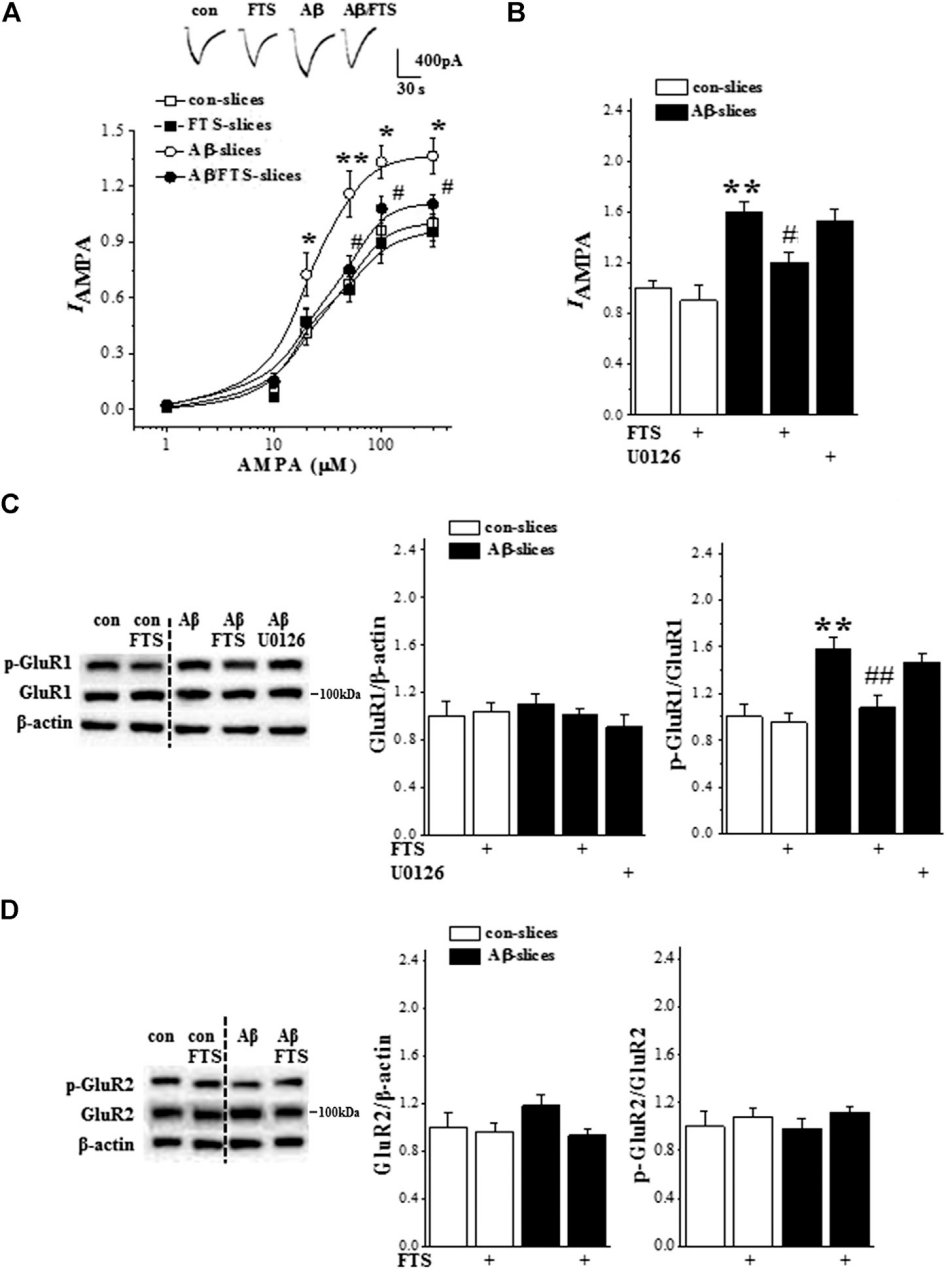
Aβ_1-42_-activated Ras enhances AMPAR function. **(A)** Evoked *I*_AMPA_ by AMPA (1–300 μM) in control slices (con), FTS-slices (FTS), Aβ_1-42_-slices (Aβ) and Aβ_1-42_/FTS-slices (Aβ/FTS). Each point represents the densities of *I*_AMPA_ normalized by a control value evoked by AMPA (300 μM). Representative traces of *I*_AMPA_ evoked by 50 μM AMPA. *p < 0.05 and **p < 0.01 vs. control slices; #*p* < 0.05 vs. Aβ_1-42_-slices (repeated-measure ANOVA, n = 6 per group). **(B)** Bars show the densities of *I*_AMPA_ (50 μM AMPA) in control slices and Aβ_1-42_-slice treated with FTS or U0126. **p < 0.01 vs. control slices; #*p* < 0.05 vs. Aβ_1-42_-slices (two-way ANOVA, n = 6 per group). **(C)** and **(D)** Bar graphs show the levels of phospho-GluR1 and phospho-GluR2 normalized by controls. **p < 0.01 vs. control slices; ##*p* < 0.01 vs. Aβ_1-42_-slices (two-way ANOVA, n = 12 per group).

Because the Aβ_1-42_-slices showed an increase in Ras activity, we further examined the response of AMPAR by whole cell patch-clamp recording (n = 6 cells/6 slices/4 mice per group). The application of AMPA in hippocampal slices evoked a dose-dependent inward current (*I*_AMPA_) in CA1 pyramidal cells (F_(5,75)_ = 136.706, *p* < 0.001; Figure 5A). The densities of *I*_AMPA_ were increased in Aβ_1-42_-slices in comparison with control slices (F_(1,10)_ = 32.619, *p* < 0.001) but showed no difference between control slices and Aβ_1-42_/FTS-slices (F_(1,10)_ = 2.680, *p* = 0.133) or FTS-slices (F_(1,10)_ = 0.198, *p* = 0.665). Furthermore, the increased density of *I*_AMPA_ in Aβ_1-42_-slices (*p* = 0.001; Figure 5B) was corrected by the addition of FTS (*p* = 0.043) rather than the MAPK kinase (MEK) inhibitor U0126 (*p* > 0.05).

GluR1/2 protein and phosphorylation (phospho-GluR1, phospho-GluR2) were examined by Western blotting (n = 12 slices/4 mice per group). The level of GluR1 protein in Aβ_1-42_-slices did not differ significantly from that in control slices (*p* > 0.05; Figure 5C), but the level of phospho-GluR1 was increased (*p* = 0.002), which was blocked by the addition of FTS (*p* = 0.009) rather than U0126 (*p* > 0.05). The Aβ_1-42_-slices did not show changes in the level of GluR2 protein (*p* > 0.05; Figure 5D) or phospho-GluR2 (*p* > 0.05).

### 3.5. Aβ_1-42_-Activated Ras Inhibits NMDAR Function via Downregulation of Src

**FIGURE 6:**
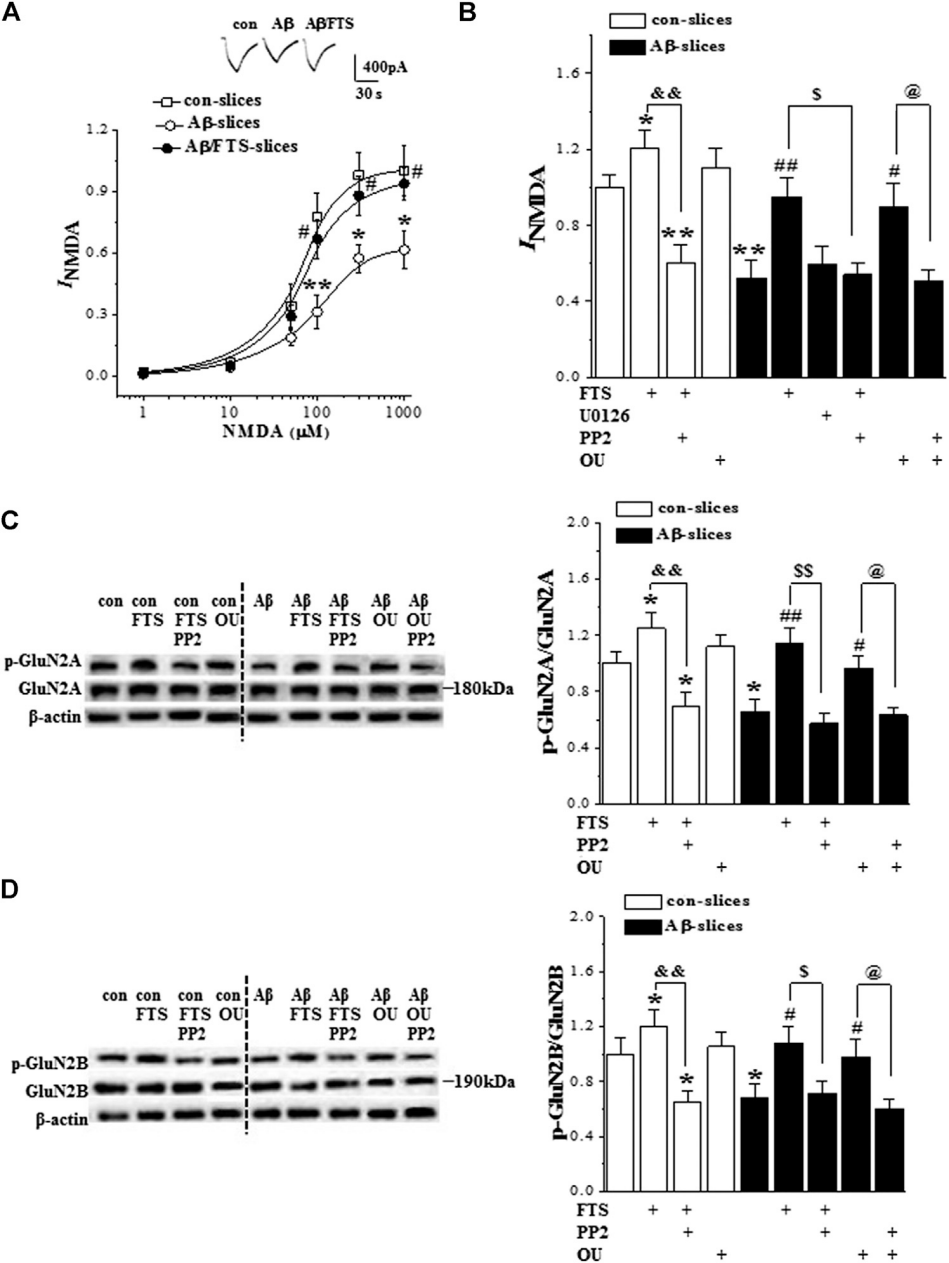
Aβ_1-42_-activated Ras reduces NMDAR function *via* down-regulation of Src. **(A)** Evoked *I*_NMDA_ by NMDA (1–1,000 μM) in in control slices (con), Aβ_1-42_-slices (Aβ) and Aβ_1-42_/FTS-slices (Aβ/FTS). Each point represents the densities of *I*_NMDA_ normalized by control value evoked by NMDA (1,000 μM). Representative traces of *I*_NMDA_ evoked by 100 μM NMDA. *p < 0.05 and **p < 0.01 vs. control slices; #*p* < 0.05 vs. Aβ_1-42_-slices (repeated-measure ANOVA, n = 6 per group). **(B)** Bars show the densities of *I*_NMDA_ (100 μM NMDA) in control slices and Aβ_1-42_-slices treated with FTS, U0126, PP2 or ouabain (OU). *p < 0.05 and **p < 0.01 vs. control slices; #*p* < 0.05 and ##*p* < 0.01 vs. Aβ_1-42_-slices; &&*p* < 0.01 vs. FTS-slices; $*p* < 0.05 vs. Aβ_1-42_/FTS-slices; @*p* < 0.05 vs. Aβ_1-42_/OU-slices (two-way ANOVA, n = 6 per group). **(C)** and **(D)** Bar graphs show the levels of hippocampal phospho-GluN2A and phospho-GluN2B normalized by controls. *p < 0.05 vs. control slices; #*p* < 0.05 and ##*p* < 0.01 vs. Aβ_1-42_-slices; and&*p* < 0.01 vs. FTS-slices; $*p* < 0.05 and $$*p* < 0.01 vs. Aβ_1-42_/FTS-slices; @*p* < 0.05 vs. Aβ_1-42_/OU-slices (two-way ANOVA, n = 12 per group).

Subsequently, we examined NMDAR activity in hippocampal CA1 pyramidal cells (n = 6 cells/6 slices/4 mice per group). In the presence of AMPAR antagonist CNQX, the application of NMDA evoked an inward current (*I*_NMDA_). The densities of *I*_NMDA_ showed a dose-dependent difference (F_(5,75)_ = 73.185, *p* < 0.001; Figure 6A). The densities of *I*_NMDA_ in Aβ_1-42_-slices were less than those in control slices (F_(1,10)_ = 31.472, *p* < 0.001), which was rescued by the addition of FTS (*p* = 0.004; Figure 6B) but not U0126 (*p* > 0.05). The protective effect of FTS on the densities of *I*_NMDA_ in Aβ_1-42_-slices was sensitive to the use of PP2 (*p* = 0.017). In addition, the density of *I*_NMDA_ in FTS-slices was higher than in the control slices (*p* = 0.026), which was corrected by the use of PP2 (*p* < 0.001). Haas et al. (2002) reported previously that ouabain binding to the Na+/K + -ATPase activated Src kinase in several different cell lines. The exposure to 5 μM ouabain for 30 min failed to increase the density of *I*_NMDA_ in control slices (*p* > 0.05). However, the treatment with ouabain could correct the density of *I*_NMDA_ in Aβ_1-42_-slices (*p* = 0.043), which was sensitive to PP2 (*p* = 0.028).

The phosphorylation of hippocampal GluN2B (phospho-GluN2B) and GluN2A (phospho-GluN2A) was further examined (n = 12 slices/4 mice per group). Compared with the controls, the levels of phospho-GluN2A (*p* = 0.011; Figure 6C) and phospho-GluN2B (*p* = 0.040; Figure 6D) were reduced in Aβ_1-42_-slices. The changes could be recovered by the use of FTS (phospho-GluN2A: *p* = 0.003; phospho-GluN2B: *p* = 0.011). The recovery of phospho-GluN2A (*p* < 0.001) or phospho-GluN2B (*p* = 0.018) in Aβ_1-42_/FTS-slices was blocked by the application of PP2. The level of phospho-GluN2A (*p* = 0.021) and phospho-GluN2B (*p* = 0.046) was increased in FTS-slices, which was sensitive to the addition of PP2 (phospho-GluN2A, *p* < 0.001; phospho-GluN2B, *p* = 0.006). Although the treatment with ouabain caused an increase in the phospho-GluN2A or phospho-GluN2B, the group when compared with control mice failed to reach the significance (*p* > 0.05). Notably, the application of ouabain could recover the levels of the phospho-GluN2A (*p* = 0.041) or phospho-GluN2B (*p* = 0.048) in Aβ_1-42_-slices, which were blocked by PP2 (GluN2A: *p* = 0.034; GluN2B: *p* = 0.029).

### 3.6. Aβ_1-42_-Activated Ras Impairs LTP and Spatial Cognition via Downregulation of Src

**FIGURE 7:**
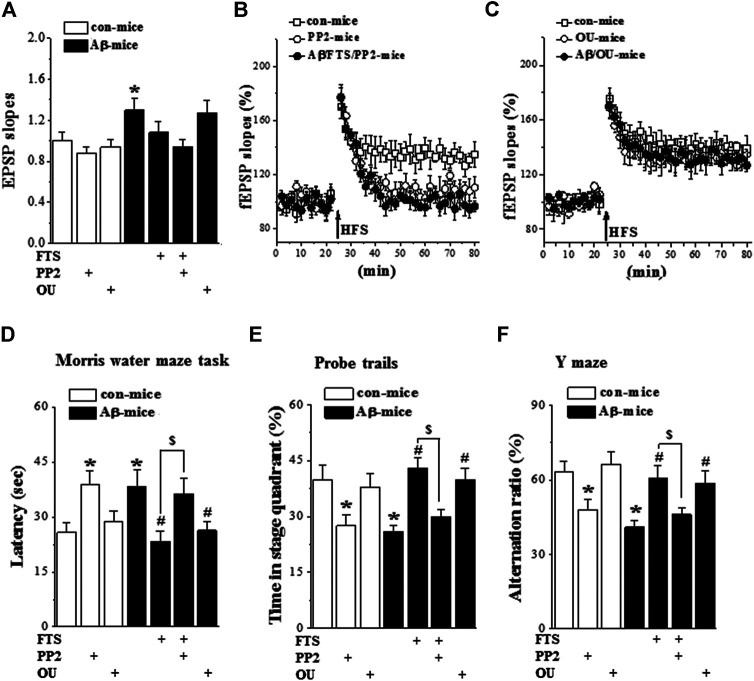
Aβ_1-42_-activated Ras impairs LTP and spatial cognition through down-regulation of Src. **(A)**–**(C)**. Input/output curve and LTP induction in control mice and Aβ_1-42_/FTS-mice treated with PP2 or ouabain. (n = 8 per group). **(D)**–**(F)**. Bar graphs represent the mean latency (sec) to reach the hidden-platform in MWM, the percentage of time spent in target quadrant in probe trails, and mean of alternation rate (%) in Y-maze task. *p < 0.05 vs. control mice; #*p* < 0.05 vs. Aβ_1-42_-mice; $*p* < 0.05 vs. Aβ_1-42_/FTS-mice (two-way ANOVA, n = 8 per group).

The chronic (three times a week) administration (i.p.) of a low dose (1 μg/kg) of ouabain in mice improves functional recovery following traumatic brain injury (Dvela-Levitt et al., 2014). To confirm whether the hyperactivation of Ras through reducing Src activity impairs LTP induction and spatial cognition, Aβ_1-42_/FTS-mice and control mice were injected (i.c.v.) with PP2; Aβ_1-42_-mice were treated (i.p.) with 1 μg/kg ouabain (Figure 1). The administration of PP2 (n = 8 slices/4 mice per group) did not alter fEPSP slopes in control mice (*p* > 0.05; Figure 7A) or Aβ_1-42_/FTS-mice (*p* > 0.05), but it suppressed the LTP induction in the both groups (Figure 7B). The fEPSP slopes were unchanged by the injection of ouabain (1 μg/kg) in control mice (*p* > 0.05) or Aβ_1-42_-mice (*p* > 0.05). Importantly, the administration of ouabain could rescue the LTP induction in Aβ_1-42_-mice (Figure 7C), although it failed to alter the LTP induction in control mice.

The administration of PP2 in Aβ_1-42_/FTS-mice (n = 8 mice per group) prevented the protective effects of FTS on the escape latency of the MWM (*p* = 0.038; Figure 7D), the swimming time in the target quadrant of the probe trial (*p* = 0.026; Figure 7E) and the alternation ratio in the Y-maze (*p* = 0.032; Figure 7F). In addition, PP2-treated control mice showed deficits in spatial cognition, such as increased escape latency in the MWM (*p* = 0.037) and reduced swimming time in the target quadrant (*p* = 0.031) or alternation ratio (*p* = 0.045). The treatment with ouabain in Aβ_1-42_-mice recovered the escape latency of the MWM (*p* = 0.034), the swimming time in the target quadrant of the probe trial (*p* = 0.041) and the alternation ratio in the Y-maze (*p* = 0.048), whereas it did not affect the spatial cognition in control mice (*p* > 0.05).

## 4. Discussion

The injection (i.c.v.) of mice with Aβ_1-42_ or acute bath application of Aβ_1-42_ in hippocampal slices could elevate hippocampal Ras activation. The oligomeric Aβ_1-42_ impaired spatial cognition and hippocampal LTP induction, which were improved by the administration of FTS. These results manifest that the oligomeric Aβ_1-42_ impairs spatial cognition *via* the hyperactivation of Ras.

In the hippocampus of Aβ_1-42_-mice, the level of Ras protein was increased by approximately 30%, while the level of Ras-GTP was elevated at least 2-fold. In hippocampal slices, the 60 min bath application of Aβ_1-42_ could enhance the activation of Ras, although it failed to alter the level of Ras protein. The results provide a clear indication that oligomeric Aβ_1-42_ not only enhances Ras expression but also stimulates Ras activation. Because Aβ binds to specific neuronal membrane APP, it has been suggested that APP is a possible receptor for Aβ (Lorenzo et al., 2000). Aβ can induce phosphorylation on APP to enhance APP proteolysis (Kirouac et al., 2017). The cytoplasmic YENPTY motif of APP is known to be a docking site for the adaptor proteins Shc and Grb2, which recruit the GEF SOS2 for activation and expression of Ras (Kirouac et al., 2017). Andras and Toborek (2013) reported that treatment with Aβ (1 μM) for 3 min caused redistribution of lipid rafts and caveola, which elevated the level of Ras-GTP. Acute treatment with Aβ_1-42_ can cascade ERK signaling in hippocampal neurons (Dineley et al., 2001). APP can positively regulate the Ras-ERK signal pathways in neuronal cell lines (Chaput et al., 2012). York et al. (1998) reported that ERK can be persistently activated by the formation of a stable upstream complex between small G proteins. In this study, either *in vivo* or *in vitro* (60 min) treatment with Aβ_1-42_ caused an increase in the levels of hippocampal ERK2 phosphorylation without changes in the expression levels, which were sensitive to Ras inhibition. The Ras isoform H-Ras may contribute to the long-lasting activation of ERK2 (Krapivinsky et al., 2003). It is conceivable that Aβ_1-42,_ through the hyperactivation of Ras, causes a rapid and sustained increase in the ERK signaling. The activation of NMDAR-mediated CaMKII can cascade the Ras-ERK signaling pathway (Carlisle et al., 2008). However, NMDAR function was downregulated in Aβ_1-42_-slices, and the blockade of NMDAR did not affect ERK2 phosphorylation. Dineley et al. (Dineley et al., 2001) reported, in the hippocampus, that chronic exposure to Aβ_1-42_ increased α7 nicotinic acetylcholine receptor (nAChR) protein, which led to chronic stimulation of ERK2. Therefore, whether Aβ_1-42_-induced hyperactivation of Ras increases ERK2 phosphorylation through enhanced *α*7nAChR function should be an interesting topic for future work.

The basal transmission of hippocampal CA3-CA1 synapses was enhanced in Aβ_1-42_-mice with no change in the capability of presynaptic glutamate release which depended on the hyperactivation of Ras rather than ERK. Postsynaptic AMPAR plays an important role in hippocampal synaptic transmission (Schwenk et al., 2014). AMPAR is synthesized dendritically and inserted into the synaptic membrane (Ju et al., 2004). The 60 min application of Aβ_1-42_ caused an obvious increase in AMPAR function and GluR1 phosphorylation, which were sensitive to Ras inhibition. The hyperactivation of Ras drives the synaptic insertion of AMPAR by triggering the phosphorylation of GluR1 at S845 and S831 (Zhu et al., 2002). Manabe et al. (2000) reported that the AMPAR synaptic responses were unchanged in CA1 pyramidal neurons of H-Ras knockout mice. Qin et al. (2005) found that the ERK1/2 phosphorylation and subsequent activation of CREB can increase AMPAR function. Enhanced ERK activity increases the expression of the GluR1 subunits (Schumann and Yaka 2009). The decline in CREB phosphorylation caused a specific downregulation of postsynaptic GluR1 (Borges and Dingledine 2001). However, the increases in AMPAR function and GluR1 phosphorylation in Aβ_1-42_-slices were insensitive to the inhibition of MEK. Furthermore, the levels of GluR1 proteins in Aβ_1-42_-slices were unchanged. A-type K+ channels have been reported to be a substrate of ERK1/2 (Schrader et al., 2006). The phosphorylation of K+ channels is known to be a primary mechanism for the ERK1/2-decreased A-type currents (Yuan et al., 2006). We observed that the inhibition of MEK failed to alter the increased fEPSP slopes in Aβ_1-42_-mice (data not shown).

NMDARs are linked to cognitive impairments in AD (Goussakov et al., 2010). The expression of GluN2A and GluN2B was downregulated in AD patient brains (Hynd et al., 2004). Aβ can induce the dysfunction of hippocampal NMDAR *via* the inactivation of GluN2B (Yin et al., 2015). The NMDAR activation is negatively regulated by the Ras signaling (Li et al., 2005). H-Ras overexpression decreases tyrosine phosphorylation of NMDAR GluN2A (Thornton et al., 2003). By inhibiting RACK1, a Ras effector protein, NMDAR currents in hippocampal neurons were increased (Yaka et al., 2002). Ras activation induces a decline in the Src autophosphorylation (Thornton et al., 2003). Src tyrosine phosphorylation positively regulates NMDAR channel activity (Sha et al., 2015). We observed that the 60 min application of Aβ_1-42_ suppressed the phosphorylation of Src and GluN2A/2B, leading to NMDAR dysfunction, which could be corrected by the inhibition of Ras or the activation of Src rather than the inactivation of MEK. The deficits in hippocampal NMDAR-dependent LTP and spatial cognition in Aβ_1-42_-mice could be rescued by the inhibition of Ras. The inhibition of Src not only reduced the phosphorylation of GluN2A/2B and NMDAR activity and depressed LTP induction and spatial cognition in control mice, but also blocked the NMDAR-dependent LTP and spatial cognition in Aβ_1-42_-mice treated with the Ras inhibitor. Moreover, the administration of Src activator at a low dose could rescue the Aβ-impaired spatial cognition and NMDAR-dependent LTP. In addition, treatment of rat brain slices with Tat-H-Ras decreased Src phosphorylation of GluN2A, and decreased the magnitude of hippocampal LTP (Thornton et al., 2003). The induction of hippocampal NMDAR-dependent LTP has been widely thought to be the basis for explicit memory formation and storage (McGaugh 2000). Thus, the results in this study give an indication that Aβ_1-42_ through suppressed Src activation blocks NMDAR-dependent LTP induction leading to impairment of spatial cognition. Ras-ERK signaling is considered to be critical for LTP induction (Patterson et al., 2010) since the inhibition of Ras or ERK blocks LTP induction (Zhu et al., 2002). Ras-ERK signaling is reported to induce the activation of L voltage-sensitive Ca2+ channel (L-VGCC) (Ekinci et al., 1999), which can enhance L-VGCC-dependent LTP (Moosmang et al., 2005).

## 5. Conclusion

The isoprenylated GTPases have been associated with the pathogenesis of AD (Ostrowski et al., 2007; Scheper et al., 2007). Ras levels are increased in brains with AD (McShea et al., 2007). In the current study, we provided the first *in vivo* and *in vitro* evidence that the Aβ_1-42_-induced abnormal increase in Ras activity may account for the impairment of NMDAR-dependent LTP induction and spatial cognition through the downregulation of Src, suggesting that targeting Ras signaling may be an effective strategy to protect hippocampal synaptic plasticity against Aβ-induced cognitive decline at early stages of AD.

## Data Availability Statement

The original contributions presented in the study are included in the article/Supplementary Material, further inquiries can be directed to the corresponding author.

## Ethics Statement

The animal study was reviewed and approved by Ethical Committee of the Nanjing Medical University.

## Author Contributions

YW performed the electrophysiological experiments and the preparation of the manuscript. ZS performed all statistical analysis. YZ undertook the western blot analysis. JY carried out the animal care and the behavioral examinations. LC and WY carried out the experimental design and revision of the manuscript.

## Conflict of Interest

The authors declare that the research was conducted in the absence of any commercial or financial relationships that could be constructed as a potential conflict of interest.

## Glossary

AD: Alzheimer’s disease
Aβ: amyloid beta
AMPAR: AMPA receptor
DMSO: dimethyl sulfoxide
EPSP: excitatory postsynaptic potential
FTS: farnesylthiosalicylic acid
HFS: high-frequency stimulation
LTP: long-term potentiation
MWM: Morris water maze
NMDAR: NMDA receptor
