# Supplementary material for: Oligomer β-amyloid Induces Hyperactivation of Ras to Impede NMDA Receptor-Dependent Long-Term Potentiation in Hippocampal CA1 of Mice
Source: Front Pharmacol. 2020 Dec 10;11:595360. doi: 10.3389/fphar.2020.595360 (PMC7848859; doi:10.3389/fphar.2020.595360)

**Supplementary information**

Frontiers in Pharmacology, section Neuropharmacology

**Oligomer β-amyloid induces hyperactivation of Ras to impede NMDA receptor-dependent long-term potentiation in hippocampal CA1 of mice**

Ya Wang^a^, Zhaochun Shi^b^,Yajie Zhang^a^, Jun Yan^c^, Wenfeng Yu^d^,**, Ling Chen^a^,*

**Entire unmodified gel example**


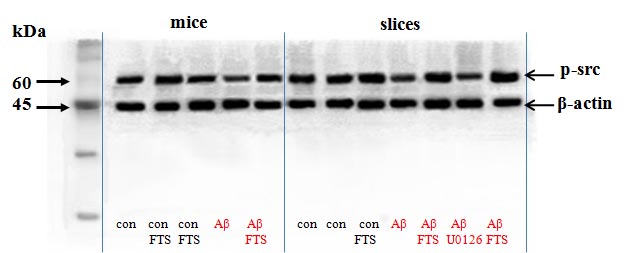


Full-length immunoblots of the hippocampal Src phosphorylation (p-Src) and β-actin (bottom) in hippocampus control mice (con), FTS-mice (con/FTS), Aβ_1-42-_mice (Aβ), Aβ_1-42_/FTS-mice (Aβ/FTS) (left side) and in hippocampal slices obtained from control slices (con), FTS-treated control slices (con/FTS), slices obtained from Aβ_1-42-_mice (Aβ), Aβ_1-42_ slices treated with FTS (Aβ/FTS) or U0126 (Aβ/U0126) (right side).

**Full scan of the entire original gels are as follows:**


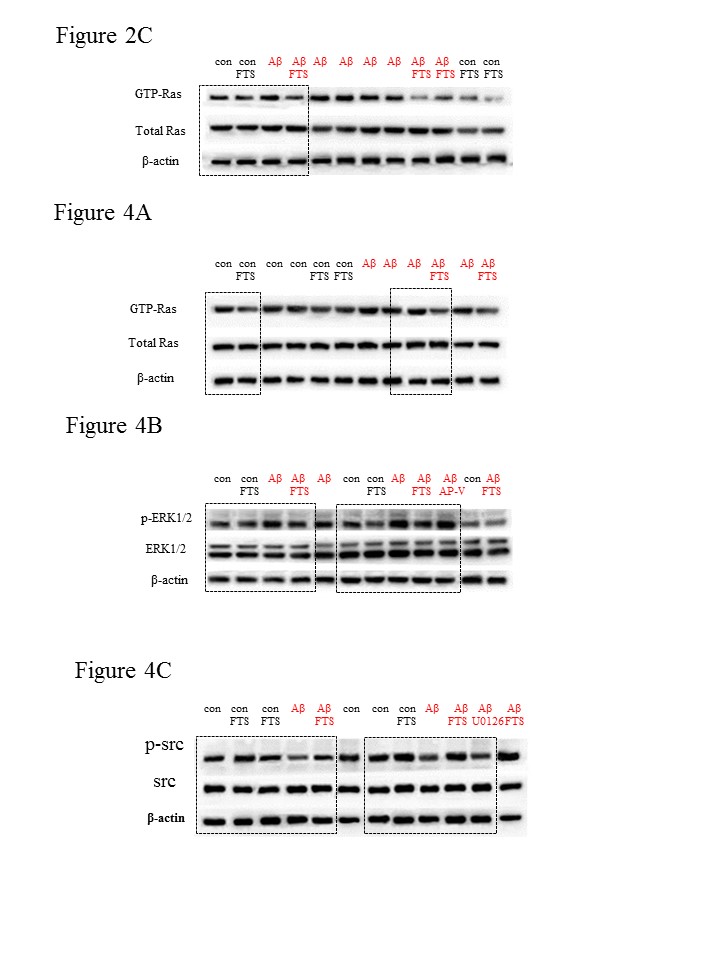


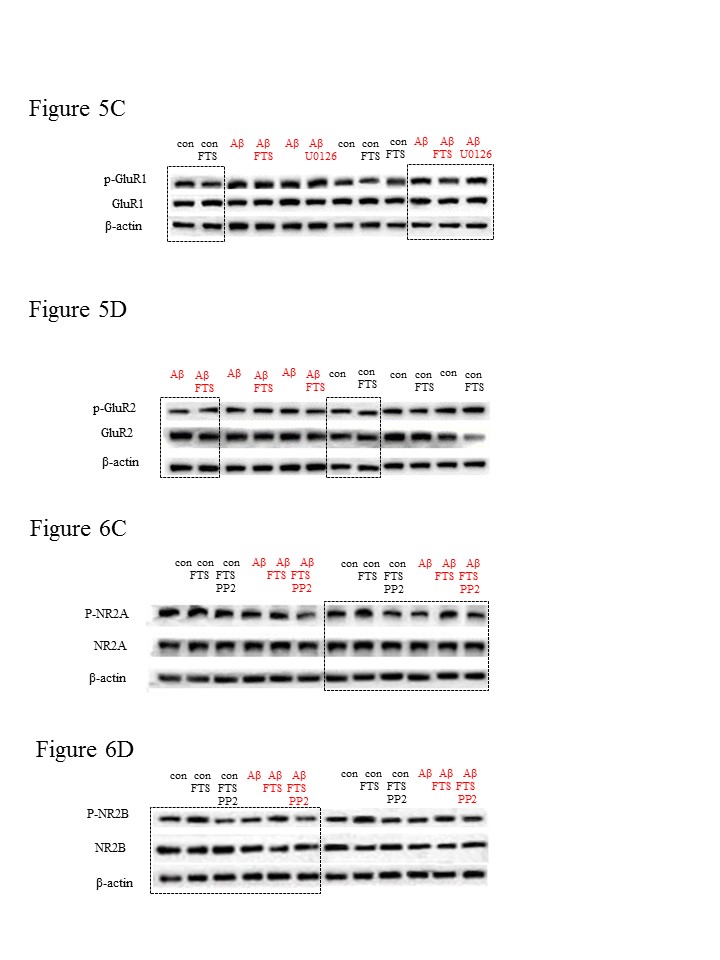

Supplement: Supplementary file 1 [file datasheet1.docx]
